# EndoClean: A Hybrid Deep Learning Framework for Automated Full-Video Boston Bowel Preparation Scale Assessment

**DOI:** 10.3390/bioengineering13030294

**Published:** 2026-03-02

**Authors:** Yan Zhu, Si-Yuan Li, Pei-Yao Fu, Zhen Zhang, Shuo Wang, Quan-Lin Li, Ping-Hong Zhou

**Affiliations:** 1 Endoscopy Center and Endoscopy Research Institute, Zhongshan Hospital, Fudan University, Shanghai 200032, China; 2 Digital Medical Research Center, School of Basic Medical Sciences, Fudan University, Shanghai 200032, China; 3 Shanghai Key Laboratory of Medical Imaging Computing and Computer Assisted Intervention, Shanghai 200032, China; 4 Shanghai Collaborative Innovation Center of Endoscopy, Shanghai 200032, China; 5 Data Science Institute, Imperial College London, London SW7 2AZ, UK

**Keywords:** artificial intelligence, colonoscopy, bowel preparation, Boston bowel preparation scale, deep learning, EndoClean

## Abstract

Background and Aims: Adequate bowel preparation is the cornerstone of high-quality colonoscopy. The Boston Bowel Preparation Scale (BBPS) is the gold standard for assessment, yet its application suffers from inter-observer variability and lacks a fully automated solution for entire video analysis. This study proposes EndoClean, a novel, fully automated deep learning framework designed to compute the full-segment BBPS score from colonoscopy videos, aiming to provide a standardized, objective, and near expert-level assessment. Methods: EndoClean integrates three distinct models: frame selection, anatomical segmentation, and BBPS scoring. Its performance was rigorously evaluated against a reference standard established by senior experts and compared with junior endoscopists. We assessed assessment precision, inter-rater agreement (quadratic weighted Kappa), and consistency across all colonic segments. Results: The EndoClean system demonstrated superior reliability, achieving a global accuracy of 97.8% for the total BBPS score, with satisfying agreement with senior experts (κ = 0.984; 95% CI: 0.976–0.989). Notably, EndoClean performed significantly better than junior endoscopists in overall BBPS agreements (κ: 0.984 vs. 0.949, *p* < 0.001) and overall accuracy (97.8% vs. 94.6%, *p* = 0.037). In segment-specific analysis, the EndoClean surpassed junior doctors particularly in the transverse colon (Accuracy: 97.5% vs. 90.4%, *p* < 0.001) and effectively reduced misclassifications in clinically ambiguous intermediate scores. For binary adequacy classification, the system achieved a sensitivity of 98.2% and a specificity of 97.3%. Conclusions: EndoClean represents a robust solution in automated quality control, demonstrating performance comparable to senior experts in bowel preparation assessment. By significantly reducing the variability seen in junior endoscopists and providing objective, full-video BBPS scoring, this framework offers a viable, standardized, and efficient solution for clinical practice and large-scale quality monitoring.

## 1. Introduction

Colorectal cancer (CRC) remains a major global health concern, ranking among the most common malignancies and leading causes of cancer-related mortality worldwide [1]. Colonoscopy is widely recognized as the gold standard for CRC screening, allowing for direct visualization of the gastrointestinal mucosa, early detection, and removal of precancerous lesions, thereby reducing CRC incidence and mortality [2,3].

Adequate bowel preparation is a prerequisite for high-quality colonoscopy, directly impacting the Adenoma Detection Rate (ADR) [4,5,6]. To standardize quality reporting, the Boston Bowel Preparation Scale (BBPS) has emerged as the most widely validated metric, requiring endoscopists to assign segment-specific scores (0–3) based on mucosal visibility. However, the clinical utility of BBPS is currently compromised by two fundamental limitations: subjectivity and inefficiency. In daily practice, scoring is highly dependent on the observer’s experience, leading to significant inter-observer variability where junior endoscopists may exhibit “optimism bias” compared to strict senior experts [7]. Furthermore, manual assessment is prone to fatigue and distraction, making it impractical for senior experts to review every procedure in high-volume centers [4,5,6]. Therefore, there is an urgent need for an automated, objective system that not only rivals expert accuracy but also reproduces BBPS-like assessments through interpretable, rule-based heuristics derived from clinical consensus, rather than inventing novel metrics, to ensure seamless integration into existing clinical workflows and provide a conservative, safety-oriented net for quality control.

Recent advances in artificial intelligence (AI) have opened new avenues for automating and standardizing colonoscopic image analysis. AI tools offer the potential to assist endoscopists in quality control and improve overall efficiency [8,9,10]. Consequently, numerous research groups have developed AI algorithms to objectively assess bowel preparation adequacy [8]. Convolutional neural networks trained on large datasets of colonoscopic images have achieved substantial agreement with human raters [6,7,8,9,10,11]. Recent advancements in AI have introduced robust automated solutions for bowel preparation assessment. Notably, Cold et al. developed the Open-Source Automatic Bowel Preparation Scale (OSABPS), which quantifies cleanliness using a pixel-level fecal-to-mucosa ratio [12]. Similarly, the e-BBPS system defined by Yu et al. has demonstrated validity for bowel preparation assessment [7]. While these metrics offer high scientific precision and correlation with adenoma detection rates (ADR), they often introduce novel quantitative indices that differ from the categorical Boston Bowel Preparation Scale (BBPS, 0–9) used in standard clinical guidelines. This discrepancy requires clinicians to ‘translate’ AI outputs into standard reports, potentially hindering seamless integration into routine workflows. Therefore, a gap remains for a fully automated system that not only processes continuous video streams but also directly replicates the standard BBPS scoring logic, serving as a ‘drop-in’ surrogate for expert assessment without altering established reporting standards.

Therefore, the development of a simple, user-friendly, and highly generalizable segmental bowel cleanliness assessment tool is significant for enhancing the standardization and clinical applicability of colonoscopy. The present study aims to design and validate such a software solution, providing a novel approach for the objective evaluation of bowel preparation quality in clinical practice.

To address this gap, we propose EndoClean, a fully automated and generalizable framework that processes colonoscopy videos to compute the BBPS score of the entire colon. EndoClean consists of two components: a segment classification model that divides the video into right, middle, and left colon segments, and a frame-level scoring model that evaluates cleanliness per BBPS criteria. By aggregating segment-wise scores, EndoClean outputs a complete BBPS score for the entire bowel, thus facilitating standardized, objective, and efficient bowel preparation assessment.

## 2. Methods

### 2.1. Datasets

This study was approved by the Ethics Committee of Zhongshan Hospital, Fudan University (Approval No. B2025-145R). The construction of EndoClean involved three distinct datasets for training the frame selection model, the frame-level scoring model, and the video-level segmentation model, respectively, followed by an independent validation set for the final system evaluation. To prevent data leakage, dataset splitting was strictly performed at the patient level, ensuring that no images or video frames from the same patient appeared in both training and validation sets.

(1) Frame Selection Dataset:

To train the model responsible for filtering out non-diagnostic frames, a specific dataset of 5670 colonoscopy images was curated. These images were annotated as either “valid” (clear visualization of mucosa) or “invalid” (blur, stool obstruction, or poor lighting). The dataset was randomly split at the patient level into a training set (4537 images) and a validation set (1133 images). This ensures the system learns to exclude low-quality inputs based on generalizable visual features rather than patient-specific artifacts.

(2) Frame-level BBPS Scoring Dataset:

The training dataset for the cleanliness assessment model was prospectively collected and annotated. A total of 35,940 colonoscopy images were curated. Senior endoscopists provided frame-level annotations according to the standard BBPS criteria (scores 0–3) or marked frames as invalid. The dataset was divided into a training set of 31,078 valid images distributed across BBPS scores 0 to 3, and a validation set of 4862 images. Invalid images were excluded from this stage to ensure the model focused solely on fine-grained cleanliness scoring, as non-diagnostic frames are pre-filtered by the Stage 1 model. Data augmentation techniques were applied to the training set to enhance model robustness against variations in endoscopic devices.

(3) Video-level Anatomical Segmentation Dataset:

The training dataset for the canatomical segmentation was prospectively collected and annotated. A total of 27,066 valid colonoscopy images were curated for this stage. Unlike the Stage 1 dataset, all images in this subset underwent a rigorous pre-screening process to exclude non-diagnostic frames.

Crucially, to address the challenge of precise anatomical localization and flexure detection, our annotation protocol was aligned with the rigorous anatomical definitions proposed in the recent research [13]. Specifically, we adopted their granular landmark criteria for identifying the hepatic flexure (visualizing the liver shadow/blueish hue) and splenic flexure (transition to descending colon). Based on these standardized landmarks, experts annotated frames into six anatomical classes (cecum, ascending colon, transverse colon, descending colon, sigmoid colon, and rectum). This alignment with state-of-the-art anatomical standards ensures that our model focuses on topologically accurate mucosal cleanliness patterns. The dataset was divided into a training set of 22,204 images and a validation set of 4862 images.

(4) System Evaluation Dataset:

To evaluate the performance of the fully integrated EndoClean framework, an independent test set consisting of 314 colonoscopy videos was collected consecutively from January 2023 to December 2024, a period subsequent to the training data collection. These videos were strictly isolated from the training and validation processes of the component models. Each video was independently scored by 5 junior endoscopists.

To establish a robust ground truth, a rigorous consensus protocol was implemented involving 5 senior endoscopists with 10 years of experience. The process adhered to the following consistency criteria: To eliminate ‘groupthink’ or authority bias, all five experts reviewed the videos independently in a blinded manner, without access to patient metadata or peer ratings. Given that BBPS scores are ordinal variables, the median value of the five experts’ scores was calculated for each colonic segment to serve as the final Ground Truth. This statistical approach was selected to minimize the impact of individual inter-observer variability and outlier ratings.

(5) Exclusion Criteria for Patients and Videos:

To ensure the quality and applicability of the dataset for automated BBPS assessment, the following exclusion criteria were applied to all collected videos:

History of Colorectal Resection: Patients with a history of partial or total colectomy were excluded, as altered anatomical structures could compromise the accuracy of the anatomical segmentation model.

Inflammatory Bowel Disease (IBD) or Severe Stenosis: Patients with active IBD (ulcerative colitis or Crohn’s disease) or severe luminal stenosis were excluded, as the mucosal features in these conditions differ significantly from routine screening populations.

Incomplete Procedures: Colonoscopies where the cecum was not reached (intubation failure) were excluded to ensure full-length video analysis.

Technical Video Defects: Videos with severe data corruption, missing segments, or extreme image artifacts that prevented human expert review were excluded.

(6) Data Preprocessing:

All colonoscopy videos were recorded using high-definition endoscopy systems (Evis Lucera Elite CV-290, Olympus Medical Systems, Tokyo, Japan) with a native resolution of 1920 × 1080 pixels. To ensure patient privacy, all protected health information (PHI), including patient ID, name, and examination date, was automatically cropped and removed from the video frames.

To balance computational efficiency with the need for temporal continuity required by the HMM-based segmentation model, videos were sampled at a rate of 5 frames per second (fps). Extracted frames were then resized to 224 × 224 pixels using bilinear interpolation to match the input dimensions of the ResNet-50 backbone. Standard normalization was applied using the mean and standard deviation of the ImageNet dataset to facilitate transfer learning.

### 2.2. Models

**Figure 1:**
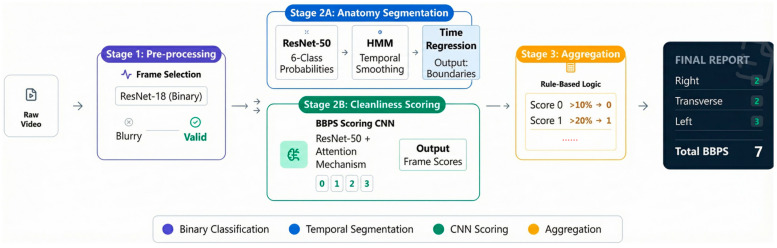
The Architecture of EndoClean. The system processes video input through a Frame Selection Model to filter invalid frames. Valid frames are processed in parallel: the Colon Segmentation Model (**top**) determines anatomical boundaries using HMM and regression, while the BBPS Scoring Model (**bottom**) evaluates cleanliness. Finally, segment-level scores are aggregated using rule-based logic to produce the total BBPS score.

In this study, we propose EndoClean, an automated system designed to compute the BBPS score from colonoscopy videos. The system consists of three distinct models to process the video in a sequence of steps, ensuring an accurate and efficient assessment of bowel preparation quality (Figure 1).

We apply a frame selection model to evaluate each frame of the colonoscopy video individually, identifying clear and assessable frames for subsequent scoring. This model is based on a convolutional neural network and performs binary classification to detect the presence of visual artifacts that may compromise scoring accuracy, such as air bubbles, blurriness, or motion blur. Only frames that are free from significant occlusions and clearly display the colonic mucosa are retained, while all others are discarded. This filtering step significantly improves the quality of inputs for the downstream models, preventing the scoring process from being affected by invalid frames. In parallel, a colon segmentation model is introduced to classify each valid frame into one of three anatomical segments: the right colon, middle colon, or left colon.

The model adopts a hybrid approach that integrates a deep neural network with a probabilistic sequence model to assign anatomical segment labels to each frame in a colonoscopy video. Unlike the other two models, this model processes entire frame sequences and formulates the segmentation task as a temporal sequence labeling problem.

In the first stage, we also trained a ResNet-50-based classifier to perform six-class frame-level classification, corresponding to six colon segments (cecum, ascending colon, transverse colon, descending colon, sigmoid colon and rectum). Once we input a sequence of frames into this model, for each frame, it will output six probabilities represent the possibility of the frame belonging to six corresponding colon segments.

Since the frame-level classification by ResNet may contain noise or abrupt transitions, we further model the task as a Hidden Markov Model (HMM) to refine the predictions by enforcing temporal continuity, thereby inferring the true underlying anatomical segment of each frame and denoising as much as possible. Specifically, we assume that the observed class probabilities produced by the ResNet are noisy manifestations of a latent, temporally smooth sequence of anatomical states. To construct the HMM, we first estimate the emission probability matrix by computing the class-wise confusion statistics of the ResNet predictions on the validation set. Then, we define a custom transition matrix that reflects the expected anatomical progression during colonoscopy, and heavily penalizes biologically implausible transitions.

Given the observation sequence, emission matrix, and transition matrix, we apply the Viterbi algorithm to compute the most likely sequence of hidden states—that is, the anatomically accurate colon segment assigned to each video frame. This HMM-based refinement enables temporally coherent, anatomically consistent segmentation, correcting noisy frame-wise predictions and producing a final sequence suitable for downstream scoring.

In detail, denote $z_{t}$ as the ResNet model’s prior estimate of the anatomical site for the t-th frame. Let $S_{t}$ denote the anatomical site assigned to the t-th frame by the HMM as the final decision, and let j index a particular anatomical site. We have:

(1) $$p\left(S_{t}|z_{t-1}\right)=\sum_{j=1}^{n}p\left(S_{t}|S_{t-1}=j\right)p\left(S_{t-1}=j|z_{t-1}\right)$$

(2) $$p\left(S_{t}|z_{t}\right)=\eta p\left(z_{t}|S_{t}\right)p\left(S_{t}|z_{t-1}\right)$$

where the term $p\left(z_{t}|S_{t}\right)$ represents the probability that the ResNet model does not misclassify, which can be obtained from the emission matrix. The term $p\left(S_{t}|S_{t-1}=j\right)$ denotes the probability of the current frame being assigned to a given site conditioned on the previous frame being site j which can be obtained from the transition matrix.

This segmentation is crucial for the BBPS scoring, as the scale requires separate cleanliness assessments for each colon segment. The segmentation model assigns each frame to its appropriate segment based on spatial features. Although the HMM refines the frame-level predictions, the resulting sequence may still exhibit boundary ambiguity or residual noise, making it difficult to directly segment the colon based on labels. Therefore, we designed a post-processing algorithm that automatically and explicitly detects the transition time points between colon segments based on the stability and trend of predicted labels across the frame sequence, thereby converting sequential information into precise temporal boundaries: the transition time points between the right-to-middle and middle-to-left colon segments. This algorithm refines the boundaries between the colon segments, which is essential for accurate scoring.

**Figure 2:**
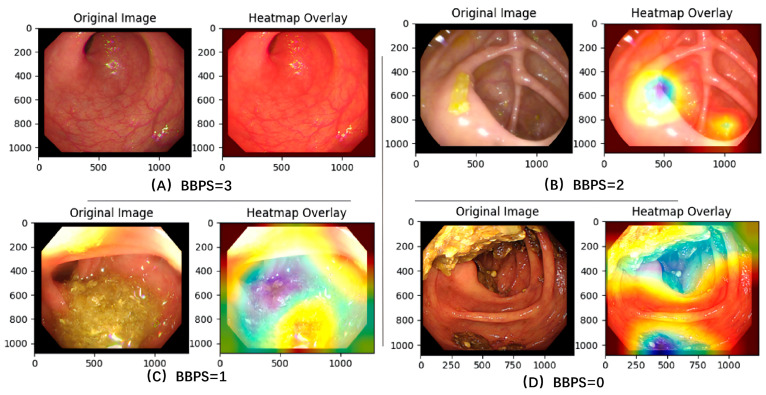
Visualization of model attention using Class Activation Mapping (CAM). (**A**,**B**) Examples of adequate bowel preparation showing minimal focal activation on clean mucosa. (**C**,**D**) Examples of inadequate preparation containing fecal residue. The heatmap overlays in (**C**,**D**) reveal that the model’s attention (blue/green regions) is strongly localized on stool and debris, demonstrating the specific identification of obscuring content.

In the final step, the selected valid frames are fed into the BBPS scoring model, which assigns a cleanliness score ranging from 0 to 3 to each frame based on the BBPS criteria. This model is built upon a deep convolutional neural network and is trained to capture key visual features. As illustrated in Figure 2, the Class Activation Mapping (CAM) visualization confirms that the model’s attention is correctly focused on discriminative regions, such as areas of mucosal visibility or obscuring stool, to generate the cleanliness score. It outputs a probability distribution over the BBPS score levels for each frame. After scoring frames individually, the system aggregates the frame-level scores within each anatomically segmented region—based on the corresponding time intervals—using a weighted average to compute segment-level scores. These are then combined to generate the final total BBPS score for the entire colonoscopy video.

The entire system operates at the frame level. It first uses Frame Selection Model to filter out valid frames, after which colon segmentation model and BBPS scoring mode run in parallel: colon segmentation model determines the temporal boundaries of the three colon segments within the video, while BBPS scoring mode assigns a cleanliness score to each valid frame. Finally, the scores of valid frames within each segment are aggregated using a weighted average to obtain the overall BBPS score for the colonoscopy video. By utilizing these three models—frame selection, colon segmentation, and BBPS scoring—EndoClean provides a fully automated, objective, and consistent method for evaluating bowel preparation quality, improving both the efficiency and accuracy of colonoscopy evaluations.

### 2.3. Algorithms

Time Points Regression for Boundary Definition

Following the probabilistic smoothing performed by the HMM (as described in Section 2.2), we obtain a coherent sequence of anatomical labels. However, to consolidate this fine-grained six-class sequence into the conventional three anatomical segments (Right, Transverse, and Left Colon) required for BBPS scoring, we need to pinpoint the exact temporal boundaries. Therefore, we designed a Time Points Regression algorithm to accurately identify the transition frames corresponding to the Hepatic and Splenic flexures.

The algorithm begins by grouping all video frames according to their HMM-predicted segment labels. Since the frame-wise predictions—even after HMM smoothing—may contain minor temporal outliers, a statistical filtering step is applied. This filtering excludes frames whose indices lie beyond two standard deviations from the mean index of the group, effectively refining the temporal distribution of each segment.

After filtering, the algorithm determines a representative median frame index for each of the six fine segments. This median serves as a temporal anchor, summarizing where that segment predominantly occurs within the video. We choose the median instead of the mean because it is more robust to outliers and accurately reflects the central distribution of labels within the temporal sequence.

Between every pair of adjacent median frames, the algorithm searches exhaustively for an optimal transition frame that best separates the two corresponding fine segments. For each candidate transition frame *m* within this interval, a composite score R(m) is calculated based on how well the frames on either side conform to their expected segment labels. Mathematically, the score is defined as:

(3) $$R\left(m\right)=\frac{L_{correct}}{L_{total}}+\frac{R_{correct}}{R_{total}}-\left|\frac{L_{correct}}{L_{total}}-\frac{R_{correct}}{R_{total}}\right|-\omega \frac{\left|L_{correct}-R_{correct}\right|}{L_{total}+R_{total}}$$

After obtaining frame-wise anatomical-site probabilities from the HMM, we identify six median frames—one for each of the six anatomical sites. We then determine five boundary points within the five segments defined by these six median frames. Let R(m) denote the likelihood score that the m-th frame within a segment serves as a boundary point, where a larger value indicates a higher likelihood. Let $L_{total}$ be the total number of frames to the left of frame m within the current segment, and let $L_{correct}$ be the number of frames on the left side that are predicted as the anatomical site corresponding to the median frame at the start of the segment. Similarly, $R_{total}$ and $R_{correct}$ are defined for the right side of frame m. We set ω = 0.8 as a balancing coefficient to discourage excessively imbalanced numbers of frames on the two sides of frame m. The scoring function consists of three components: the first two terms encourage high classification accuracy on both sides, and the third term penalizes large discrepancies in accuracy between the two sides. The candidate frame with the highest score is selected as the optimal transition point between the two fine-grained segments.

Clinical Landmark Identification 

To accurately delineate the three broad colon segments, our model leverages the six fine-grained anatomical classes (Cecum, Ascending Colon, Transverse Colon, Descending Colon, Sigmoid Colon, and Rectum). By applying the temporal boundary detection algorithm described above, we identify the five anatomical transition frames. Among them, the second and third boundaries—corresponding to the Hepatic Flexure (between Ascending and Transverse Colon) and Splenic Flexure (between Transverse and Descending Colon)—are selected as the clinically meaningful transition points. This two-stage strategy (HMM smoothing followed by Time Points Regression) leverages temporal context to precisely localize key anatomical landmarks, thereby reducing frame-level noise and enhancing the accuracy of the downstream BBPS assessment.

BBPS estimation 

Once the temporal boundaries are established, the system integrates the frame-level BBPS predictions (generated in parallel as described in Section 2.2) to estimate the overall score for each segment. We developed a rule-based aggregation algorithm that prioritizes the detection of inadequate bowel preparation. Specifically, using the two identified transition frames (Hepatic and Splenic flexures), the colonoscopy video is divided into three anatomically meaningful regions. For each segment, the algorithm collects the BBPS scores of all valid frames and computes the frequency of each score level (0, 1, 2, and 3). Based on these statistics, the final segmental score is determined according to the following rules:

- If the proportion of frames scored as 0 exceeds 10%, indicating a substantial presence of unprepared mucosa, the segment score is set to 0;
- Otherwise, if the proportion of frames scored as 1 exceeds 20%, suggesting generally poor preparation, the segment score is set to 1;
- If neither condition is met, the average BBPS score of all frames in the segment is computed and rounded to the nearest integer to obtain the final score.

From a clinical perspective, these thresholds reflect a ‘safety-oriented’ scoring strategy. Score 0 represents solid stool or obstruction that prevents visualization, which poses the highest risk for missed lesions; therefore, a stricter threshold (10%) was applied to ensure high sensitivity for inadequate preparation. Score 1 represents minor staining or liquid that allows some visualization, justifying a slightly more lenient threshold (20%). This proportion-based quantification method aligns with recent benchmarks in automated bowel preparation assessment, ensuring that the aggregated video-level score accurately reflects the worst-case scenario observed within the segment.

After computing the segmental scores for all three regions, the system aggregates them to derive the total BBPS score for the entire colonoscopy video, providing a quantitative and objective assessment of bowel preparation quality for clinical evaluation.

### 2.4. Implementation Details

Frame Selection Model

The model is built upon the ResNet-18 architecture. We adopted the pre-trained weights from ImageNet to initialize the model. The dataset described above (4537 training and 1133 validation images) was used to fine-tune the network. During training, we applied data augmentation techniques, including color jitter, random affine, and random rotation. The model was optimized using the Adam optimizer with a learning rate of 1 × 10^−3^ and a batch size of 512. The weighted binary cross-entropy loss function was used to address class imbalance. The model checkpoint exhibiting the lowest loss on the validation set was selected for integration into the final EndoClean system.

Colon Segmentation Model

In the first stage, the ResNet-50 architecture was adapted for six-class classification and initialized with ImageNet pre-trained weights. The model was trained using the 22,204 images from the segmentation training set. Data augmentation techniques were applied to enhance robustness. The model was trained using categorical cross-entropy loss, optimized with the Adam optimizer (learning rate 1 × 10^−3^, weight decay 1 × 10^−2^). Training was monitored using the validation set (9790 images), and the optimal model was retained to generate observation probabilities for the subsequent HMM stage.

BBPS Scoring Model

The BBPS Scoring Model employs a customized architecture integrating ResNet-50 with a dedicated attention mechanism. The model was trained from scratch on the dataset of 31,078 images (Training Set) described in the dataset section. It outputs a probability distribution across the BBPS score levels for each frame. Performance was monitored on the validation set of 4862 images to prevent overfitting and ensure generalization capability.

### 2.5. Statistical Analysis

Continuous variables were expressed as mean ± standard deviation (SD) or median with interquartile range (IQR), depending on the data distribution. Categorical variables were presented as frequencies and percentages. Differences in baseline characteristics were evaluated using the Student’s *t*-test or Mann–Whitney U test for continuous variables, and the Chi-square test or Fisher’s exact test for categorical variables.

The primary performance metrics for the AI system and endoscopists included sensitivity, specificity, accuracy, positive predictive value (PPV), and negative predictive value (NPV). 95% confidence intervals (CIs) for all performance metrics were calculated using either the Wilson score interval or the bootstrap method (1000 resamples).

Inter-rater agreement between the AI system/junior endoscopists and the gold standard (senior experts) was assessed using the quadratic weighted Kappa (κ) coefficient and the Intraclass Correlation Coefficient (ICC, two-way random effects model, absolute agreement). The strength of agreement was interpreted as follows: 0–0.20 (slight), 0.21–0.40 (fair), 0.41–0.60 (moderate), 0.61–0.80 (substantial), and 0.81–1.00 (almost perfect).

To statistically compare the performance differences between the AI system and junior endoscopists, McNemar’s test was used for paired nominal data (accuracy, sensitivity, and specificity). The difference in Kappa coefficients between the two groups was tested for statistical significance using a bootstrap-based test for equality of dependent kappa coefficients. The relationship between the AI scores and the gold standard was further analyzed using Pearson correlation coefficients (r) and Bland–Altman analysis to assess systematic bias.

All statistical tests were two-sided, and a *p*-value of <0.05 was considered statistically significant. Statistical analyses were performed using Python (scikit-learn library, version 1.0.2) and SPSS software (version 21.0, IBM Corp., Armonk, NY, USA).

## 3. Results

### 3.1. EndoClean Achieves High Reliability in Total BBPS Scoring Compared to Junior Endoscopists

The validation phase included a total of 314 colonoscopy videos, representing a diverse range of bowel preparation qualities. To establish a robust evaluation benchmark, five senior endoscopists generated the reference standard, while five junior endoscopists independently scored the same dataset to provide a comparative baseline for human performance.

The baseline characteristics of the 314 validation videos are summarized in Table 1. The dataset included a balanced distribution of gender (51.0% male) and a mean patient age of 58.4 years. The most common indication was screening (47.5%), and the median total BBPS score was 6, with 52.5% of patients having adequate bowel preparation (Table 1).

For the primary outcome—the total BBPS score—the EndoClean system demonstrated high levels of agreement with the senior experts. As detailed in Table 2, the EndoClean achieved a quadratic weighted Kappa (QWK) of 0.984 (95% CI 0.976–0.989), which was significantly higher than the 0.949 (95% CI 0.928–0.964) achieved by the junior endoscopists (*p* < 0.001). This superiority in agreement was further corroborated by the Intraclass Correlation Coefficient (ICC), where the EndoClean scored 0.984 compared to 0.949 for the junior group, indicating good consistency with the gold standard.

Beyond agreement metrics, the EndoClean system exhibited greater precision in score estimation. The Mean Absolute Error (MAE) for the EndoClean was 0.105, substantially lower than the 0.258 observed in the junior group. Similarly, the Root Mean Square Error (RMSE) was reduced from 0.584 for junior doctors to 0.324 for the EndoClean, reflecting the model’s stability in minimizing large deviations from the expert consensus. Although the differences in sensitivity (98.2% vs. 96.4%) and specificity (97.3% vs. 92.6%) did not reach statistical significance, the EndoClean’s overall accuracy of 0.978 was statistically superior to the 0.946 accuracy of junior doctors (*p* = 0.037). These findings suggest that the EndoClean system can replicate expert-level judgment for the overall bowel cleanliness assessment more reliably than less experienced clinicians.

### 3.2. EndoClean Performed Significantly Better than Junior Doctors in Segment-Level BBPS Scoring

When analyzing performance across specific anatomical segments, the EndoClean model consistently matched or surpassed the precision of junior endoscopists, with the most notable advantages observed in the transverse colon.

In the right colon, a critical segment for adenoma detection, the EndoClean maintained consistent reliability. It achieved a satisfying sensitivity of 1.000 and a quadratic weighted Kappa of 0.957. While the agreement level was comparable to junior doctors (Kappa 0.947, *p* = 0.500), the EndoClean demonstrated significantly higher overall accuracy (0.994 vs. 0.965, *p* = 0.011), ensuring that cleanliness in this high-risk segment is assessed with high accuracy (Table 3).

The performance gap was most pronounced in the transverse colon, historically the most challenging segment for consistent human rating due to variable visualization. Here, the EndoClean showed a statistically significant improvement over the junior group, maintaining a high Kappa of 0.947 compared to 0.835 for junior doctors (*p* = 0.001). Furthermore, the EndoClean achieved an accuracy of 0.975, which was markedly superior to the 0.904 accuracy recorded by the junior group (*p* < 0.001). This indicates that the EndoClean system is more robust against the visual ambiguities that often confound trainees in the middle segment.

In the left colon, both the EndoClean and junior doctors showed strong agreement with senior experts (Kappa 0.936 vs. 0.944, *p* = 0.629). However, the EndoClean exhibited significantly higher sensitivity (0.996 vs. 0.936, *p* < 0.001) and overall accuracy (0.984 vs. 0.946, *p* = 0.009). This suggests that while junior doctors are generally competent in the left colon, the EndoClean is more effective at consistently detecting adequate preparation with comparable specificity (95.1% vs. 97.5%, *p* = 0.404) (Table 3).

To further validate the clinical reliability of the system, we assessed predictive values and systematic bias. In the binary classification of bowel preparation adequacy (Adequate vs. Inadequate), the EndoClean system demonstrated superior predictive capabilities compared to junior endoscopists. The EndoClean achieved a global Positive Predictive Value (PPV) of 97.6% and a Negative Predictive Value (NPV) of 98.0% (vs. 93.5% and 95.8% for juniors). Crucially, in the right colon—a high-risk region for missed adenomas—the EndoClean attained a good NPV, ensuring that inadequate preparations in this validation set were effectively flagged (Table 4).

**Figure 3:**
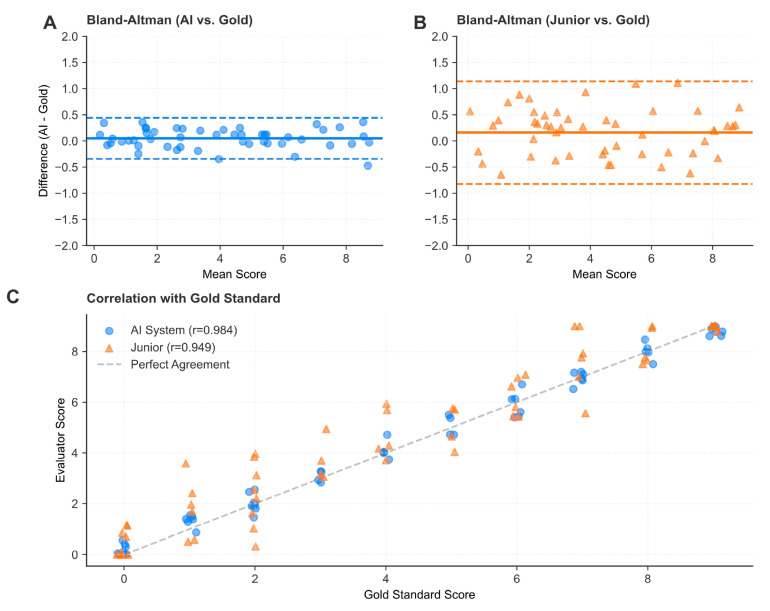
Agreement and correlation analysis of the total Boston Bowel Preparation Scale (BBPS) score. (**A**,**B**) Bland–Altman plots. Comparison of agreement between the EndoClean system and the Gold Standard (**A**), and between Junior Endoscopists and the Gold Standard (**B**). The solid lines represent the mean difference (bias), and the dashed lines represent the 95% limits of agreement. The EndoClean system demonstrates a negligible bias (0.054) and narrower limits of agreement compared to Junior Endoscopists (bias 0.061). (**C**) Correlation scatter plot. The relationship between the Gold Standard scores (x-axis) and the evaluated scores (y-axis). The EndoClean system (circles, r = 0.984) shows tighter clustering along the diagonal line of good agreement compared to Junior Endoscopists (triangles, r = 0.949), indicating superior consistency across the full range of bowel preparation quality.

**Figure 4:**
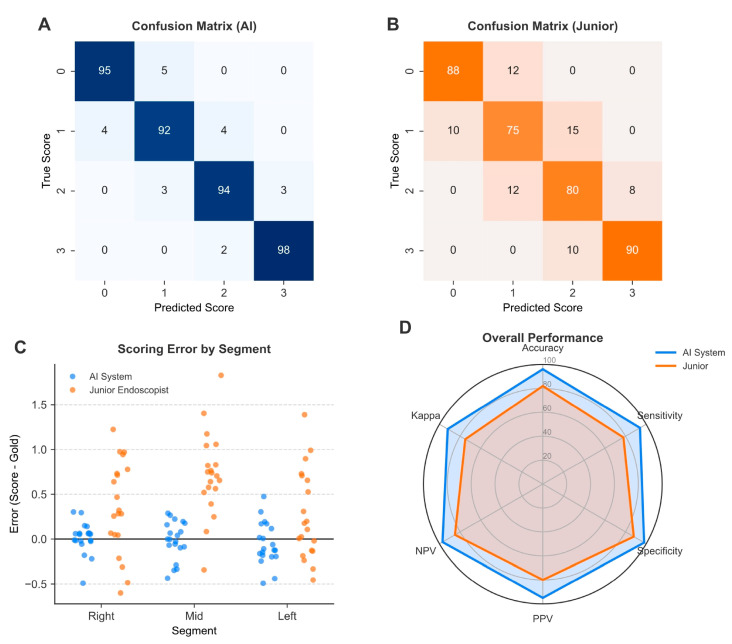
Diagnostic accuracy and error analysis by colonic segment. (**A**,**B**) Normalized confusion matrices. Heatmaps displaying the classification accuracy for segment-level BBPS scores (0–3). Darker blue indicates higher concordance. The EndoClean system (**A**) maintains high accuracy across all scores, whereas Junior Endoscopists (**B**) show increased misclassification rates for intermediate scores (1 vs. 2). (**C**) Distribution of scoring errors by segment. Scatter plot showing the deviation from the Gold Standard (y-axis: Evaluator Score minus Gold Standard). The zero line represents good agreement. Junior Endoscopists (triangles) exhibit a distinct positive shift in the Mid (Transverse) colon, indicative of “optimism bias” due to visualization difficulties, whereas the EndoClean system (circles) remains centered. (**D**) Radar chart of performance metrics. A multi-dimensional comparison showing that the EndoClean system outperforms Junior Endoscopists across most global performance metrics, particularly in Accuracy and Kappa. While Junior Endoscopists showed comparable sensitivity in specific segments, the AI system demonstrates a more balanced diagnostic profile with superior specificity and overall reliability.

Regarding scoring objectivity, the EndoClean showed a stronger linear relationship with the gold standard (Pearson r = 0.984) than junior doctors (r = 0.949). Analysis of systematic error (Bias) revealed a notable divergence in the transverse colon: junior endoscopists exhibited a significant positive bias (+0.092), indicating a tendency to overestimate cleanliness in this anatomically complex segment. In contrast, the EndoClean maintained a slight negative bias (−0.035), reflecting a more rigorous and conservative scoring strategy that minimizes the risk of score inflation (Figure 3 and Figure 4; Table 4).

Although the sensitivity for identifying a BBPS score of 0 in the right colon was 76.9% (Table 5). Since both score 0 and score 1 represent ‘inadequate’ bowel preparation, these granular misclassifications did not affect the system’s clinical decision-making capability. Consequently, the system maintained a good Negative Predictive Value for discriminating between adequate and inadequate preparation in the right colon (Table 4).

### 3.3. EndoClean Reduces Misclassifications of Intermediate BBPS Scores—Key to Clinical Decision-Making

The clinical utility of an automated scoring system depends heavily on its ability to distinguish between intermediate scores, particularly BBPS score 1 (poor) and score 2 (good), as this threshold dictates the adequacy of bowel preparation and subsequent surveillance intervals. Performance analysis stratified by individual BBPS scores revealed that the EndoClean’s advantage over junior endoscopists was most pronounced in these clinically ambiguous categories (Figure 4).

In the right colon, accurate identification of adequate preparation is paramount for polyp detection. The EndoClean model achieved an accuracy of 97.1% in correctly classifying BBPS score 2.

In the transverse colon, where visual obstruction often complicates scoring, the EndoClean demonstrated robustness for BBPS score 1, achieving 96.8% accuracy. This substantial gap highlights the EndoClean’s ability to maintain consistent criteria in challenging segments where human observers are prone to error.

For the extreme scores (0 and 3), the EndoClean performed consistently well across all segments. For instance, in the left colon, the sensitivity for BBPS score 3 was 98.8%. These findings collectively suggest that while junior doctors are proficient at recognizing obvious extremes of cleanliness, the EndoClean system provides significant added value by reducing human variability in the interpretation of borderline cases, which are often the most pivotal for clinical decision-making.

## 4. Discussion

### 4.1. Standardizing Quality Control: EndoClean as a Reliable Surrogate for Expert Assessment

Our study demonstrates that a deep learning-based system can autonomously evaluate bowel preparation quality with an accuracy that rivals senior experts and significantly surpasses junior endoscopists. While previous studies have explored AI for bowel preparation, our findings are pivotal in validating that an automated system can effectively replicate the Boston Bowel Preparation Scale (BBPS)—the global standard for quality reporting—across diverse colon segments. The core implication of this work is not merely that AI is “accurate,” but that it offers a viable, standardized solution to the persistent problem of inter-observer variability in clinical practice. Furthermore, the EndoClean system demonstrated superior discriminative ability, particularly in the transverse colon, where it achieved an AUC of 0.983 compared to 0.880 for junior endoscopists, highlighting its robustness in challenging visual conditions. While comparing AI against other AI models is valuable for technical benchmarking, our study prioritized the comparison against junior endoscopists to address a specific clinical urgency: the ‘Optimism Bias’ and inter-rater variability. By demonstrating that EndoClean significantly outperforms junior staff and aligns with senior experts (Table 2), we validate the system’s primary clinical utility—acting as an automated quality control guardrail in daily practice where senior supervision is limited.

### 4.2. The Efficiency-Accuracy Trade-Off: A Worthwhile Exchange

A critical interpretation of our findings requires contextualizing the statistical comparison against the “Gold Standard” of senior expert consensus. Although the EndoClean model’s overall accuracy (97.8%) was marginally lower than the consensus-derived standard, this difference must be weighed against the realities of clinical workflows [14]. In high-volume endoscopy centers, the capacity for senior experts to review every procedure is severely limited. Consequently, the negligible statistical gap between the AI and a perfect human expert is a worthwhile exchange for the immense benefits of automation, consistency, and immediate availability. Unlike human operators, whose judgment is susceptible to fatigue, distraction, and time constraints, the AI system functions as an “always-on” expert consultant. It provides a stable, objective metric that remains constant regardless of procedure volume or time of day. By delivering performance that is robust enough to replace the variable assessments of less experienced endoscopists, the system elevates the baseline quality of care without imposing additional oversight burdens on senior staff [15].

### 4.3. Clinical Safety and Objectivity: The Value of Conservative Scoring

Beyond absolute accuracy, the clinical reliability of EndoClean relies heavily on its “anatomical awareness.” Addressing the concern that global high accuracy might mask segmentation failures (e.g., in homogeneously prepared colons), our data offers a robust rebuttal based on clinical heterogeneity. Clinical experience confirms that bowel preparation is rarely uniform; specifically, the Right Colon frequently presents with lower scores due to solid stool retention compared to the Left Colon. If our segmentation model failed to correctly identify the Right Colon, the system would erroneously attribute the cleaner features of distal segments to it, causing a collapse in agreement with expert scores. The high segment-specific agreement observed in our results (Right Colon Accuracy: 99.4%) therefore serves as a strong indirect validation that the model is correctly localizing anatomical segments despite their visual differences.

To achieve this, our hybrid architecture employs a Hidden Markov Model (HMM) to enforce temporal consistency. Unlike static image classification, the HMM leverages the irreversible sequential nature of the withdrawal phase (Cecum to Rectum). This allows the system to maintain accurate anatomical localization via probabilistic inference, even when visual landmarks in individual frames are ambiguous. This design prioritizes sequence-level integrity—which is critical for calculating aggregate BBPS scores—over pixel-perfect boundary detection, aligning with the practical needs of clinical quality reporting.

Regarding scoring safety, our results showed that junior endoscopists tended to overestimate bowel cleanliness in the transverse colon (positive bias), a phenomenon likely attributable to the “optimism bias” often seen when human graders confront complex folds or partially obscured views. The EndoClean, conversely, displayed a negligible to slightly negative bias. In the context of quality control, a conservative scorer is clinically safer than a lenient one. By strictly adhering to BBPS criteria and refusing to avoid score inflation for marginal preparations, EndoClean encourages a higher standard of mucosal washing. Furthermore, the system’s high Negative Predictive Value (NPV), particularly the 100% NPV achieved in the right colon, provides a robust safety net. In clinical practice, the cost of falsely labeling an inadequate preparation as “adequate” is high, as it may lead to missed lesions and delayed surveillance. The EndoClean’s performance ensures that when a segment is deemed “inadequate”, it reliably warrants intervention, thereby supporting safer clinical decision-making.

### 4.4. Why Mimicking BBPS Matters: Seamless Integration over Novelty

In the rapidly evolving landscape of medical AI, automated systems frequently introduce novel, proprietary metrics to quantify bowel cleanliness. For instance, the recently published OSABPS by Cold et al. employs a sophisticated “fecal ratio”, calculating the proportion of fecal pixels relative to mucosal pixels. Similarly, the e-BBPS system defines a cleanliness score ranging from 0 to 20 based on frame-wise validity [12], It is crucial to distinguish the validation strategy of EndoClean from these systems. Because metrics like the 0–20 scale or pixel ratios are new to the medical community, validating them directly against Adenoma Detection Rate (ADR) was a statistical necessity to prove their clinical relevance. In contrast, EndoClean is designed to replicate the “Real BBPS”—the categorical standard already enshrined in clinical guidelines. Since the strong correlation between adequate BBPS scores and higher ADR is a settled medical fact established by landmark studies, our validation focuses on the fidelity of the reproduction. By proving that EndoClean reproduces the senior expert’s judgment with high precision (r = 0.984), the system inherits the established clinical validity of the BBPS itself.

Furthermore, regarding the definition of the “Gold Standard”, our study adopts the rigorous annotation methodology standard in medical computer vision. We utilized a multi-expert consensus protocol involving independent blinded review followed by conflict adjudication. This approach mitigates individual subjectivity and establishes a “Ground Truth” that represents the collective intelligence of senior specialists. While direct validation against ADR remains the ultimate goal for future multi-center randomized controlled trials, this study provides the essential technical validation: proving that AI can function as an objective “Bias Corrector”.

This design philosophy prioritizes “clinical interoperability”. Unlike the pixel-ratio approach, which treats the colon as a collection of pixels, EndoClean’s architecture mimics the endoscopist’s cognitive process to render a categorical judgment. This ensures that the output is instantly interpretable: a “Score 2” from EndoClean means exactly the same as a “Score 2” from a senior expert. By eliminating the “Optimism Bias” of junior endoscopists and standardizing quality reporting to an expert level, EndoClean offers a decisive advantage for large-scale deployment without disrupting the established clinical workflow.

## 5. Limitations and Future Directions

Despite the promising results, our study is not without limitations. First, as a retrospective single-center study, there is an inherent risk of selection bias. The training and validation datasets, while large, may not fully capture the visual diversity of bowel preparations encountered in different populations or with different endoscopic equipment manufacturers. Multi-center validation is the necessary next step to confirm the robustness of our algorithm across varying clinical settings.

Second, regarding the technical architecture, our current framework employs a hybrid CNN-HMM. While the HMM effectively leverages the fixed anatomical sequence to smooth predictions, we acknowledge that this approach may not capture long-range temporal dependencies as effectively as emerging deep learning architectures. As suggested by recent literature, Video Transformers represent a promising direction for future work, with their ability to model complex temporal dynamics via self-attention mechanisms potentially improving segmentation precision, particularly in procedures with erratic camera movements or loops.

Concurrently, the core cleanliness assessment is primarily based on frame-level feature extraction using 2D-CNNs. Although this approach achieves high accuracy by aggregating scores across segments, it may not fully capture complex spatiotemporal dynamics—such as the rapid movement of fluid or debris that an endoscopist dynamically visualizes. Future iterations will therefore aim to incorporate 3D-CNNs or Transformer-based architectures to better leverage spatiotemporal continuity, enabling a more robust, real-time dynamic assessment that mirrors the continuous cognitive process of a human expert.

Finally, while we have established that the EndoClean correlates well with expert BBPS scoring, the ultimate litmus test for any bowel preparation assessment tool is its association with clinical outcomes, specifically the Adenoma Detection Rate (ADR). Future studies should investigate whether AI-derived BBPS scores are as predictive of ADR as human-derived scores. If the EndoClean system can reliably flag inadequate preparation that leads to missed lesions, its value shifts from a mere documentation tool to a critical quality assurance instrument that directly impacts patient safety.

## 6. Conclusions

In conclusion, this study validates a deep learning system that effectively automates the assessment of bowel preparation quality using the widely accepted BBPS standard. By achieving a performance level comparable to senior experts and superior to junior endoscopists, the EndoClean system offers a pragmatic solution to the challenge of inter-observer variability. It provides a “qualified and usable” standard of assessment that, while requiring a negligible compromise in absolute accuracy compared to top-tier experts, delivers substantial improvements in consistency, efficiency, and availability. By embedding the standard BBPS metrics into an automated workflow, our approach bridges the gap between expert guidelines and daily practice, paving the way for standardized quality control in colonoscopy on a global scale.

## Figures and Tables

**Table 1:** Baseline Characteristics of the Colonoscopy Videos in the Validation Set.

Characteristic	Value (N = 314)
Patient Demographics	
Age, mean ± SD, years	58.4 ± 12.3
Male sex, n (%)	160 (51.0%)
Procedure Indications, n (%)	
Screening	149 (47.5%)
Surveillance	99 (31.5%)
Diagnostic	66 (21.0%)
Bowel Preparation Quality	
Total BBPS Score, median (IQR)	6 (5–8)
Adequate Preparation (Score ≥ 6), n (%)	52.5% (165/314)
Segmental Scores, mean ± SD	
Right Colon	2.1 ± 0.8
Transverse Colon	2.0 ± 0.9
Left Colon	2.3 ± 0.7

**Table 2:** Overall Inter-rater Agreement and Scoring Performance: EndoClean Model vs. Junior Endoscopists.

Metric	EndoClean Model (95% CI)	Junior Endoscopists (95% CI)	*p* Value
Agreement with Gold Standard			
Quadratic Weighted Kappa	0.984 (0.976–0.989)	0.949 (0.928–0.964)	<0.001
ICC	0.984 (0.980–0.990)	0.949 (0.940–0.960)	<0.001
Pearson Correlation (r)	0.984	0.949	0.500
Error Metrics			
Mean Absolute Error (MAE)	0.105	0.258	—
Root Mean Square Error (RMSE)	0.324	0.584	—
Accuracy			
Accuracy	97.8% (97.0–98.6%)	94.6% (93.3–95.9%)	0.037
Sensitivity	98.2% (97.2–99.2%)	96.4% (95.0–97.8%)	0.310
Specificity	97.3% (96.0–98.6%)	92.6% (90.5–94.7%)	0.062

Note: ICC, Intraclass Correlation Coefficient. *p* values compare EndoClean vs. Junior Endoscopists.

**Table 3:** Comparison of Performance Metrics by Colonic Segment.

Metric	EndoClean Model (95% CI)	Junior Endoscopists (95% CI)	*p* Value
Right Colon			
Quadratic Kappa	0.957 (0.936–0.977)	0.947 (0.922–0.967)	0.500
Sensitivity	100.0% (100.0–100.0%)	95.6% (94.2–97.0%)	0.500
Specificity	98.2% (96.9–99.4%)	98.2% (96.9–99.4%)	1.000
Accuracy	99.4% (98.9–99.8%)	96.5% (95.5–97.5%)	0.011
Transverse Colon			
Quadratic Kappa	0.947 (0.906–0.981)	0.835 (0.771–0.888)	0.001
Sensitivity	96.4% (95.1–97.6%)	98.6% (97.8–99.4%)	0.126
Specificity	100.0% (100.0–100.0%)	71.6% (67.0–76.2%)	0.050
Accuracy	97.5% (96.6–98.3%)	90.4% (88.8–92.1%)	<0.001
Left Colon			
Quadratic Kappa	0.936 (0.910–0.958)	0.944 (0.922–0.964)	0.629
Sensitivity	99.6% (99.2–100.0%)	93.6% (92.0–95.2%)	<0.001
Specificity	95.1% (92.7–97.4%)	97.5% (95.8–99.2%)	0.404
Accuracy	98.4% (97.7–99.1%)	94.6% (93.3–95.9%)	0.009

Sensitivity: Represents for Binary Adequacy Sensitivity, calculated based on the ability to correctly identify inadequate bowel preparation (Total BBPS < 6 or any segment score < 2).

**Table 4:** Comparison of Clinical Utility, Discriminative Ability, and Systematic Bias between EndoClean and Junior Endoscopists.

Segment	Metric	EndoClean (AI)	Junior Endoscopists
Right Colon	PPV	99.0% (204/206)	99.0% (195/197)
	NPV	100.0% (108/108)	92.3% (108/117)
	AUC	0.993	0.976
	Bias	0.022	0.000
Transverse	PPV	100.0% (211/211)	88.9% (216/243)
	NPV	92.2% (95/103)	95.8% (68/71)
	AUC	0.983	0.880
	Bias	−0.035	+0.092
Left Colon	PPV	98.3% (232/236)	99.1% (218/220)
	NPV	98.7% (77/78)	84.0% (79/94)
	AUC	0.984	0.963
	Bias	0.067	−0.032
Total Score	PPV	97.6% (162/166)	93.5% (159/170)
	NPV	98.0% (145/148)	95.8% (138/144)
	AUC	0.990	0.974
	Bias	0.054	0.061

**Table 5:** Performance of AI Model per Score across Segments.

Segment	Score	Accuracy (95% CI)	Sensitivity (95% CI)	Specificity (95% CI)	PPV (95% CI)
Right	0	97.5% (95.1–98.7%)	76.9% (57.9–89.0%)	99.3% (97.5–99.8%)	90.9% (72.2–97.5%)
	1	96.8% (94.2–98.3%)	95.2% (88.4–98.1%)	97.4% (94.4–98.8%)	93.0% (85.6–96.8%)
	2	97.1% (94.6–98.5%)	97.5% (93.8–99.0%)	96.7% (92.6–98.6%)	96.9% (93.0–98.7%)
	3	97.8% (95.5–98.9%)	93.0% (81.4–97.6%)	98.5% (96.3–99.4%)	90.9% (78.8–96.4%)
Mid	0	99.4% (97.7–99.8%)	100.0% (34.2–100.0%)	99.4% (97.7–99.8%)	50.0% (15.0–85.0%)
	1	96.8% (94.2–98.3%)	97.8% (92.5–99.4%)	96.4% (93.0–98.2%)	91.9% (84.9–95.8%)
	2	97.8% (95.5–98.9%)	95.7% (91.3–97.9%)	100.0% (97.6–100.0%)	100.0% (97.6–100.0%)
	3	99.0% (97.2–99.7%)	96.6% (88.3–99.0%)	99.6% (97.8–99.9%)	98.2% (90.7–99.7%)
Left	0	99.0% (97.2–99.7%)	66.7% (35.4–87.9%)	100.0% (98.8–100.0%)	100.0% (61.0–100.0%)
	1	97.5% (95.1–98.7%)	94.4% (86.6–97.8%)	98.3% (95.8–99.4%)	94.4% (86.6–97.8%)
	2	93.0% (89.6–95.3%)	88.5% (82.4–92.7%)	97.0% (93.1–98.7%)	96.3% (91.7–98.4%)
	3	94.6% (91.5–96.6%)	98.8% (93.6–99.8%)	93.0% (89.0–95.7%)	84.0% (75.6–89.9%)

Note: CI = Confidence Interval; PPV = Positive Predictive Value. The 95% CIs were calculated using the Wilson score interval method.

## Data Availability

The original contributions presented in the study are included in the article, further inquiries can be directed to the corresponding authors.

## References

[1] Prashanth R., Tagore S., Adam B. (2019). Epidemiology of colorectal cancer: Incidence, mortality, survival, and risk factors. Gastroenterol. Rev..

[2] Antonio Z.G.-G., Enrique Q. (2023). Role of colonoscopy in colorectal cancer screening: Available evidence. Best Pract. Res. Clin. Gastroenterol..

[3] Rex D.K. (2025). Colonoscopy Remains an Important Option for Primary Screening for Colorectal Cancer. Dig. Dis. Sci..

[4] Hassan T., Muhammad Umar K., Binita S., Fady E., Usman Ali P., Nanda P., Sara A., Aiyi Z., Ahmed B., Hafsa A. (2019). Evaluation of the combined effect of factors influencing bowel preparation and adenoma detection rates in patients undergoing colonoscopy. BMJ Open Gastroenterol..

[5] Liwen Y., Huizhen X., Qiucheng L., Wen W., Zhifeng W., Xia T., Chaijie L., Hang Y., Chenxia Z., Lihui Z. (2024). Validation of artificial intelligence–based bowel preparation assessment in screening colonoscopy (with video). Gastrointest. Endosc..

[6] Ji Young L., Audrey H.C., William K., James R., Brian C.J., Michael B.W. (2022). Artificial intelligence for the assessment of bowel preparation. Gastrointest. Endosc..

[7] Ji Young L., Jooyoung P., Hyo Jeong L., Hana P., Eun Hyo J., Kanggil P., Ji Eun B., Dong-Hoon Y., Seung Wook H., Namkug K. (2024). Automatic assessment of bowel preparation by an artificial intelligence model and its clinical applicability. J. Gastroenterol. Hepatol..

[8] Xirong X., Jiahao L., Jianwei Q., Benfang F., Tao H., Shichun F., Jinjie S., Zhenming G. (2025). The Application Value of an Artificial Intelligence-Driven Intestinal Image Recognition Model to Evaluate Intestinal Preparation before Colonoscopy. Br. J. Hosp. Med..

[9] Singh T., Handa P., Goel N., Kaur H., Jakhetiya V., Goyal P., Khanna P., Raman B., Kumar S. (2024). Automated BBPS Scoring in Colonoscopy: A Comparative Analysis of Pre-trained Deep Learning Architectures. Computer Vision and Image Processing.

[10] Cold K.M., Ali A., Konge L., Bjerrum F., Lovat L., Ahmad O. (2025). Bowel preparation assessment using artificial intelligence: Systematic review. Endosc. Int. Open.

[11] Buijs M.M., Ramezani M.H., Herp J., Kroijer R., Kobaek-Larsen M., Baatrup G., Nadimi E.S. (2018). Assessment of bowel cleansing quality in colon capsule endoscopy using machine learning: A pilot study. Endosc. Int. Open.

[12] Cold K.M., Heen A., Vamadevan A., Vilmann A.S., Konge L., Rasmussen M., Svendsen M.B.S. (2025). Development and validation of the Open-Source Automatic Bowel Preparation Scale. Gastrointest. Endosc..

[13] Song Y., Zhang Z., Wang R., Zhong L., Cai C., Chen J., Zhou Y., Wang X., Li Z., Yang L. (2025). CAS-Colon: A Comprehensive Colonoscopy Anatomical Segmentation Dataset for Artificial Intelligence Development. Sci. Data.

[14] Rex D.K., Anderson J.C., Butterly L.F., Day L.W., Dominitz J.A., Kaltenbach T., Ladabaum U., Levin T.R., Shaukat A., Achkar J.P. (2024). Quality indicators for colonoscopy. Gastrointest. Endosc..

[15] Feng L., Xu J., Ji X., Chen L., Xing S., Liu B., Han J., Zhao K., Li J., Xia S. (2023). Development and validation of a three-dimensional deep learning-based system for assessing bowel preparation on colonoscopy video. Front. Med..

